# Contact Dermatitis From Exposure to Virginia Creeper (Parthenocissus quinquefolia): A Deviation From the Saying “Leaves of Three, Let It Be”

**DOI:** 10.7759/cureus.86240

**Published:** 2025-06-17

**Authors:** Drew M Frase, Susan Bannon

**Affiliations:** 1 Department of Internal Medicine, Western Michigan University Homer Stryker M.D. School of Medicine, Kalamazoo, USA

**Keywords:** allergic contact dermatitis, allergy, contact dermatitis, dermatology, phytodermatitis, toxicodendron, type iv hypersensitivity, virginia creeper

## Abstract

Phytodermatitis is often caused by contact with certain plants and may be recognized by the common saying,* "leaves of three, let it be". * However, lesser-known plants, such as Virginia creeper (*Parthenocissus quinquefolia*), can also cause significant contact dermatitis (CD).

A 54-year-old female physician with a history of poison ivy allergy presented with a pruritic, erythematous, blistering rash with linear streaks on her face, neck, and arms following exposure to Virginia creeper plants in her backyard. Symptoms began 48 hours post-exposure, exhibiting linear streaks and scattered plaques suggestive of allergic contact dermatitis (ACD). The patient self-treated with topical betamethasone, diphenhydramine, ibuprofen, and petroleum jelly. Symptoms significantly improved within one week, with complete resolution by one month. Removal of Virginia creeper plants from her backyard prevented further recurrence.

Clinicians and the public should be aware that Virginia creeper, despite its five-leaf configuration, can cause phytodermatitis that is very similar to poison ivy. Recognition and identification of plant-induced dermatitis are significant for effective management and prevention.

## Introduction

Contact dermatitis (CD) encompasses a broad differential of inflammatory skin conditions caused by exposure to allergens or irritants [1]. Phytodermatitis, a subset of CD resulting from plant exposures, can present with varying degrees of erythema, vesiculation, and pruritus, depending on the causative agent and individual sensitivity [2]. In North America, the majority of allergic phytodermatitis cases are attributed to members of the Anacardiaceae family, particularly the *Toxicodendron *genus, which includes poison ivy, poison oak, and poison sumac [3]. Public education campaigns have emphasized recognizing and avoiding these plants, often summarized by the adage “leaves of three, let it be” [2].

However, not all plant-induced dermatitis fits neatly into this pattern. Virginia creeper (*Parthenocissus quinquefolia*), a commonly encountered vine featuring five leaflets, is generally not recognized by the public as a potential cause of allergic or irritant dermatitis. Although primarily associated with irritant contact dermatitis (ICD) due to mechanical irritation by oxalate crystals [4,5], CD involving Virginia creeper has been sporadically documented [2], thus highlighting the novelty of our case report. The distinction between allergic and irritant mechanisms is crucial, as allergic phytodermatitis involves a type IV hypersensitivity response mediated by T cells [6], whereas ICD results from direct cytotoxic effects [1,2]. Misidentification of Virginia creeper as benign may delay appropriate management and increase the risk of repeated exposure and symptom exacerbation.

The following case report describes a middle-aged woman who developed a classic allergic contact dermatitis (ACD) pattern following exposure to Virginia creeper. This report aims to raise awareness about less commonly recognized botanical causes of CD by illustrating the clinical course, differential diagnosis, and therapeutic response. Increased knowledge among clinicians and the public may contribute to more accurate diagnosis, timely management, and improved patient outcomes.

## Case presentation

A 54-year-old female physician presented with a pruritic, blistering rash on her face, neck, and arms. The rash presented with linear streaks and scattered plaques, likely due to the exposure pattern and subsequent scratching. She reported that 48 hours prior to the onset of symptoms, she had removed Virginia creeper vines from a tree in her backyard. She initially felt what she thought were mosquito bites but subsequently developed intense pruritus and erythematous lesions on her forehead, nose, upper lip, face, neck, and both arms. The patient had a known allergy to poison ivy, for which she had previously required oral steroids. However, on this occasion, she had not been exposed to poison ivy. She had previously suspected that Virginia creeper might have been responsible for similar reactions in the past, but had always attributed them to poison ivy.

**Figure 1:**
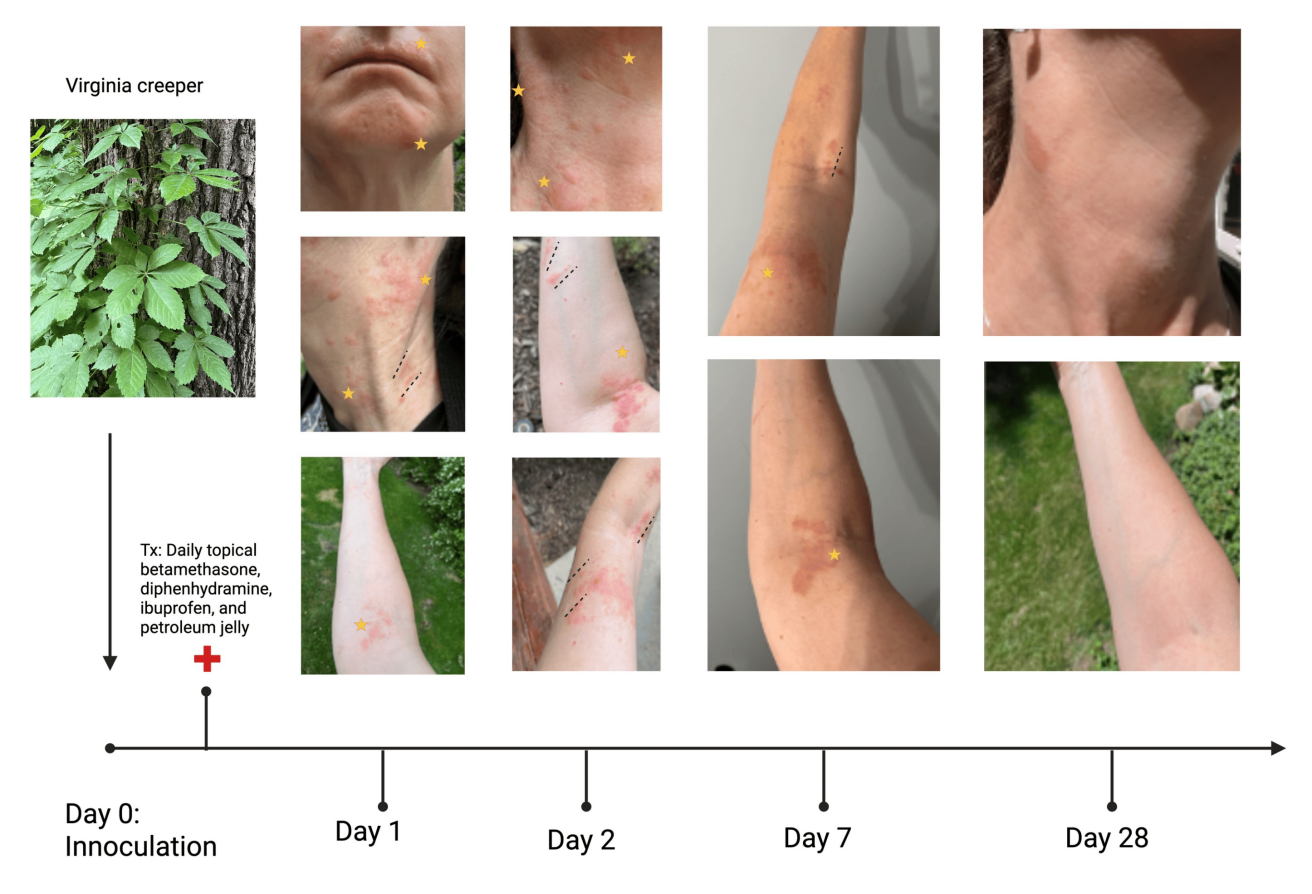
Timeline from inoculation to symptom resolution Images show the five-leaved Virginia creeper plant along with the progression of erythematous, blistering plaques on the patient's chin, neck, and arms bilaterally. Plaques are arranged in linear streaks, representing exposure, and a scattered pattern likely due to scratching. Prior to day 1 post inoculation, the patient began treating her rash and symptoms with daily betamethasone, diphenhydramine, ibuprofen, and petroleum jelly. Gradual resolution occurred by seven days, with complete resolution by day 28 post inoculation. Gold stars represent sites of dermatologic rash. Dashed lines (----) mark linear streaks. Tx: treatment

Her medical history included asthma and eczema, and her regular medication was pimecrolimus topical PRN. She self-treated the current rash with prescription betamethasone cream, ibuprofen, diphenhydramine, and petroleum jelly. She was experiencing severe itching, but only minimal blistering and weeping compared to her usual poison ivy reactions. The patient continued to experience symptoms for one week, at which point the rash began to resolve. The rash was completely resolved by day 28 (Figure 1).

Investigations

Written informed consent was obtained from the patient for publication of her case, clinical details, and accompanying images. The patient did not undergo any lab tests or imaging studies for medical workup. No biopsy or allergen patch testing was performed. In our case, the diagnosis was clinical and based on history and exam. A thorough dermatological physical examination showed large areas of raised, erythematous plaques arranged linearly on the face, neck, and arms, consistent with CD. As a physician, the patient was comfortable with conservative management in lieu of invasive testing.

Differential diagnosis

The differential diagnosis for the patient's rash included several possible dermatological conditions. ACD due to poison ivy (phytodermatitis) was initially considered due to the patient's history of plant allergy, recent episode of yard work, and the presentation of erythematous, pruritic lesions on exposed skin. However, the lack of known exposure and the mild blistering and weeping patterns helped rule it out. ACD due to Virginia creeper was still on the differential, given the patient’s known exposure and dermatological presentation. ACD due to other irritants was also considered, but the patient had no known exposure to cosmetics, fragrances, or metals, such as nickel or cobalt.

Prurigo from insect bites was initially suspected, given the pruritic, erythematous nature of the rash, considering the patient felt what she thought were mosquito bites. However, the progression to a widespread rash and linear configuration was inconsistent with typical insect bite reactions. We considered airborne CD, but our patient’s lesions were more localized to contact areas rather than in a diffuse aerosolized pattern. Atopic dermatitis was also considered due to the patient's history of eczema, but the acute nature of the symptoms and apparent trigger made this diagnosis less probable. Interestingly, systematic review and meta-analysis show no association between atopic dermatitis and the development of contact sensitization [7]. We also excluded occupational allergen exposure, given the patient’s known exposure while gardening at home. The specific pattern and patient's history suggested phytodermatitis as a more likely diagnosis.

**Figure 2:**
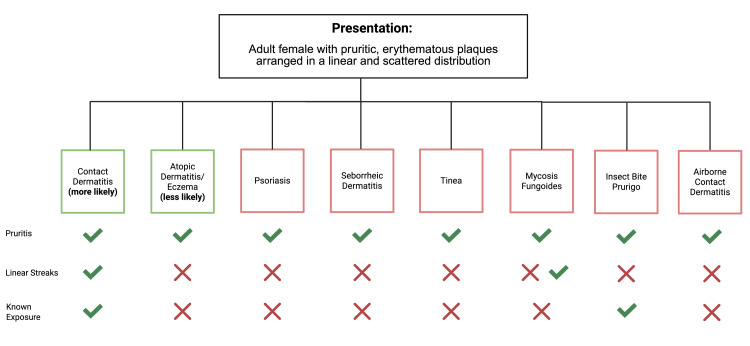
Differential diagnosis of an erythematous, pruritic rash in an adult female Given the patient's presentation with pruritic, erythematous plaques arranged linearly on the head, neck, and arms, we started with a broad differential. Using the patient's past medical history of asthma/eczema and known plant exposure, we narrowed our differential to either contact dermatitis or atopic dermatitis.

Less likely diagnoses on the differential for a woman presenting with an erythematous, pruritic rash included seborrheic dermatitis, psoriasis, tinea infection, and mycosis fungoides (Figure 2). Seborrheic dermatitis was excluded due to a lack of greasy, oily skin. The patient’s rash also exhibited no scaling, thus making psoriasis, tinea infection, and mycosis fungoides unlikely. Notably, in rare cases, mycosis fungoides appears in a linear, Blaschkoid presentation. However, acute onset within 48 hours post gardening and localization to exposed areas favored an external contact source. Ultimately, the diagnosis of phytodermatitis from Virginia creeper was made based on the patient's previous history and characteristic pattern of rash in linear streaks.

Treatment

The treatment plan for the patient's phytodermatitis involved a combination of pharmacological and non-pharmacological approaches. The patient self-treated with topical betamethasone cream upon symptom onset, which was applied to reduce inflammation and alleviate itching. Currently, recommendations for poison ivy dermatitis include high-to-super high potency topical corticosteroids like betamethasone or clobetasol [8]. Additionally, she took oral ibuprofen to manage pain and further reduce inflammation. To address the severe itching and assist with sleep, the patient used diphenhydramine, an antihistamine. Interestingly, antihistamines are not routinely utilized to treat poison ivy CD, and pruritus in these cases is not driven by histamine [9,10]. Petroleum jelly was applied to affected areas to prevent further irritation. This regimen led to a gradual improvement of pruritus and erythema over the course of several days. Follow-up at day 28 confirmed full resolution of the rash, and the patient reported no recurrence of symptoms or further complications. Resolution in less than a month with no further monitoring parallels the natural course of CD secondary to poison ivy exposure [4]. The remaining Virginia creeper plants were removed from the patient’s yard to prevent future exposure.

Outcome and follow-up

The patient experienced gradual improvement over one week, with the rash resolving without significant complications. Follow-up at two weeks showed improvement in symptoms and gradual resolution of the lesions, with full dermatological and symptomatic recovery by day 28. The patient reported no recurrence of symptoms.

## Discussion

**Figure 3:**
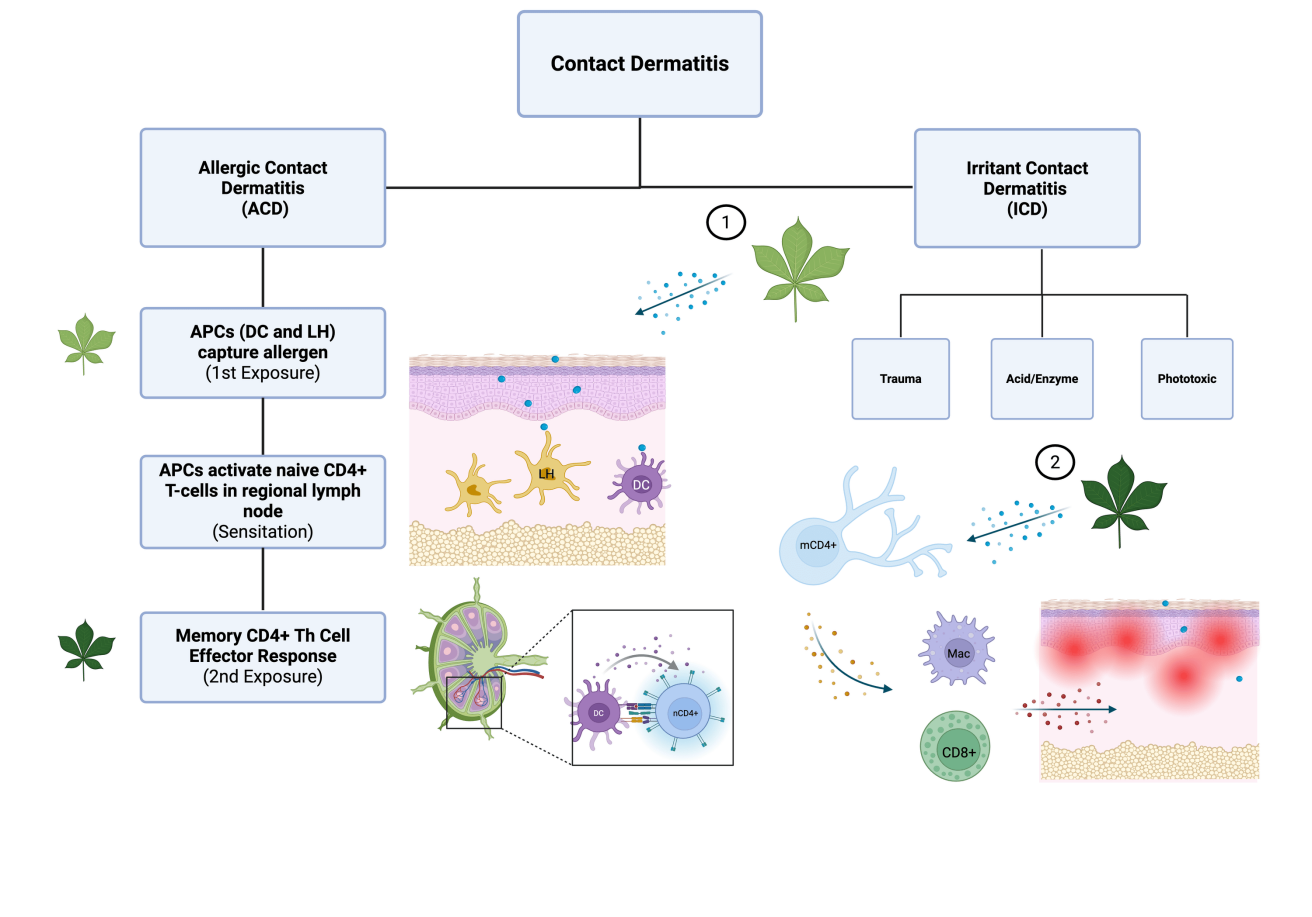
Allergen sensitization in allergic contact dermatitis (CD) CD can be classified as either allergic (ACD) or irritant (ICD). ICD is non-immune mediated, occurring through direct effects on the skin through either physical trauma, acid/enzyme release, or via chemicals with phototoxicity (mechanism not shown). In ACD, plant allergens are picked up by antigen-presenting cells (APCs), either Langerhans cells (LH) or dermal dendritic cells (DCs). APCs then present allergens to naive CD4+ T-cells (nCD4+) in regional lymph nodes and drive activation and differentiation into CD4+ memory T-cells (mCD4+), which completes sensitization. Upon re-exposure, CD4+ memory T-cells immediately activate effector cytotoxic T-cells (CD8+) and macrophages, which release inflammatory cytokines into the skin that produce symptoms. Image credit: Drew M. Frase; created with BioRender (Science Suite Inc., Toronto, Canada); Sources: [6, 12]

Phytodermatitis from Virginia creeper is a relatively underreported condition, with most cases of plant-induced CD attributed to poison ivy or similar species [2]. Similar studies have been reported from inoculation with poison ivy [4, 11]. In ACD, allergen-specific CD4 T-helper cells exhibit a type IV hypersensitivity reaction [2, 6]. In a primary exposure-sensitization phase, small plant allergens within plant resin enter the stratum corneum layer, wherein they can bind to self-proteins and form hapten-protein complexes [6]. These foreign complexes are recognized by antigen-presenting cells (APCs), which include dendritic cells and dermal Langerhans cells, and presented on MHC class II receptors [6,12]. Once activated, the APCs navigate to regional lymph nodes where they can present the digested allergen proteins to naive CD4+ T-cells. Antigen presentation activates naive D4+ T-cells and drives their differentiation into antigen-specific CD4+ memory T-cells (Figure 3) [6, 12]. Subsequent exposure to the same antigen stimulates memory CD4+ T-cells to navigate towards the inoculated site and produce regional inflammation via activation of CD8+ cytotoxic T-cells and macrophages, also known as the elicitation phase [6,12].

On the other hand, during ICD, skin is directly damaged by the cytotoxic effects of a specific irritant; it is not immune-mediated and requires no sensitization phase [1]. ICD can further be classified by three primary mechanisms: chemical insult, mechanical trauma, and phototoxic insult (Figure 3) [2]. Considering the rash’s spread beyond initial exposure sites and temporal association with plant exposure, the mechanism of the Virginia creeper is more consistent with ACD. A limitation of our study includes a lack of true causality, as ACD cannot be definitively confirmed without a biopsy or allergen-specific patch/prick testing. Histologic features of spongiosis and eosinophilic infiltrate would have further supported an allergic mechanism. However, in our case, the patient opted for conservative management over invasive testing.

Multiple compounds have been identified in other species that are known to cause contact dermatitis. For example, *Toxicodendron* species like poison ivy release the chemical allergen urushiol, while *Parthenocissus *species release irritant oxalate crystals [4,5]. Both urushiol allergens and oxalate crystal irritants cause CD [3, 10]. While Virginia creeper-induced ACD is clinically plausible based on the patient’s symptoms and timeline, it remains rare and chemically unconfirmed, with no definitive allergen isolated to date.

## Conclusions

To date, there is a paucity of studies reporting episodes of CD secondary to Virginia creeper exposure. This novel case emphasizes the importance of accurate plant identification and public education regarding the risks associated with Virginia creeper. Similar cases in the literature are scarce, and the primary differentiator from poison ivy CD is known exposure to a five-leaved plant rather than the well-known three-leaved poison ivy plant, highlighting the need for increased awareness and reporting of such incidents. Notably, the deviation from the "leaves of three, let it be" saying can lead to misidentification and improper management, prolonging patient suffering.
